# Antimicrobial Activity of Quasi-Enantiomeric *Cinchona* Alkaloid Derivatives and Prediction Model Developed by Machine Learning

**DOI:** 10.3390/antibiotics10060659

**Published:** 2021-05-31

**Authors:** Alma Ramić, Mirjana Skočibušić, Renata Odžak, Ana Čipak Gašparović, Lidija Milković, Ana Mikelić, Karlo Sović, Ines Primožič, Tomica Hrenar

**Affiliations:** 1 Faculty of Science, University of Zagreb, 10000 Zagreb, Croatia; alma.ramic@chem.pmf.hr (A.R.); ana.mikelic@chem.pmf.hr (A.M.); karlo.sovic@chem.pmf.hr (K.S.); 2 Faculty of Science, University of Split, 21000 Split, Croatia; mirskoc@pmfst.hr (M.S.); rodzak@pmfst.hr (R.O.); 3 Ruđer Bošković Institute, 10000 Zagreb, Croatia; ana.cipak@irb.hr (A.Č.G.); lidija.milkovic@irb.hr (L.M.)

**Keywords:** quaternary cinchonidines, quaternary cinchonines, antimicrobial activity, cytotoxicity, ROS, activity/PES model, machine learning

## Abstract

Bacterial infections that do not respond to current treatments are increasing, thus there is a need for the development of new antibiotics. Series of 20 *N*-substituted quaternary salts of cinchonidine (CD) and their quasi-enantiomer cinchonine (CN) were prepared and their antimicrobial activity was assessed against a diverse panel of Gram-positive and Gram-negative bacteria. All tested compounds showed good antimicrobial potential (minimum inhibitory concentration (MIC) values 1.56 to 125.00 μg/mL), proved to be nontoxic to different human cell lines, and did not influence the production of reactive oxygen species (ROS). Seven compounds showed very strong bioactivity against some of the tested Gram-negative bacteria (MIC for *E. coli* and *K. pneumoniae* 6.25 μg/mL; MIC for *P. aeruginosa* 1.56 μg/mL). To establish a connection between antimicrobial data and potential energy surfaces (PES) of the compounds, activity/PES models using principal components of the disc diffusion assay and MIC and data towards PES data were built. An extensive machine learning procedure for the generation and cross-validation of multivariate linear regression models with a linear combination of original variables as well as their higher-order polynomial terms was performed. The best possible models with predicted *R*^2^(CD derivatives) = 0.9979 and *R*^2^(CN derivatives) = 0.9873 were established and presented. This activity/PES model can be used for accurate prediction of activities for new compounds based solely on their potential energy surfaces, which will enable wider screening and guided search for new potential leads. Based on the obtained results, *N*-quaternary derivatives of *Cinchona* alkaloids proved to be an excellent scaffold for further optimization of novel antibiotic species.

## 1. Introduction

**Figure 1:**
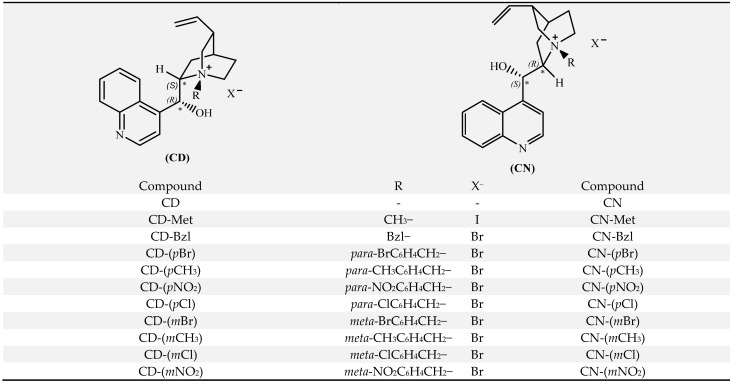
Structures of cinchonidine (**CD**) and cinchonine (**CN**) compounds. Absolute configurations, opposite in quasi-enantiomers at positions 8 and 9, are noted; (8S,9R) in **CD** and (8R,9S) in **CN**.

Bacterial drug resistance is one of the major problems in public health worldwide. Reports from different health organizations make claims that antibacterial resistance is responsible for more than 35,000 deaths in the United States and about 33,000 deaths in European Union, annually [1,2]. A lot of effort has been put into the research and development of antibacterial agents against emerging new bacterial strains. In the search for new classes of antibacterial agents, various groups of alkaloids were extensively researched and employed as scaffolds, such as metronidazole, quinolones, indoles, and others [3]. *Cinchona* alkaloids are natural products isolated from the bark of the *Cinchona* tree and the most known are quinine (**Q**), quinidine (**QD**), cinchonine (**CN**), and cinchonidine (**CD**). The structure of these alkaloids consists of a bulky quinuclidine ring with a vinyl side chain, an aromatic quinoline ring, and a hydroxyl group at C9. They have five chiral centers (N1, C3, C4, C8, and C9) and two of them, C8 and C9, can have different absolute configuration in **Q**/**QD** and **CN**/**CD** pairs, so these diastereomers are often called quasi-enantiomers. Structures of **CN** and **CD** are presented in Figure 1.

Because of their availability and interesting properties, derivatives of *Cinchona* alkaloids have various applications in all fields of chemistry, for example as chiral resolving agents or chiral stationary phases for chromatographic separation, as a chiral catalyst or chiral ligands in asymmetric synthesis [4,5,6]. They also possess a wide range of biological activity, which is not surprising since for decades, **Q** was used for the treatment of malaria [7]. Besides anti-malarial activity, they have anti-inflammatory, anti-arrhythmic, anti-proliferative, and insecticidal activity, among others [8,9,10]. Qi et al. performed experiments in vitro which showed that **CN** could induce apoptosis and reduce the proliferation of cancer cells, and experiments in animals showed that it could suppress tumor growth in mice [11]. These findings were confirmed by Jo et al., who showed that **CN** inhibits osteoclast differentiation and promotes osteoblast differentiation [12]. Another group designed and synthesized indocinchona alkaloids, an alkaloid with merged **QN** and indole rings. They identified one of them, azaquindol, as a novel class of autophagy inhibitors, which play a crucial role in cancer and degenerative diseases [13]. **QN** scaffold was used for creating active nanostructured coatings with the ability to release antibacterial compounds against *Escherichia Coli* [14]. Optochin is a *Cinchona* alkaloid derivative that poses highly selective antibacterial activity towards *Streptococcus pneumoniae* and it is used as a laboratory standard for differentiation of *Streptococcus pneumoniae* from other streptococci [15]. Aldrich et al. recently prepared a series of new optochin derivatives, and one of them showed increasing activity toward multidrug-resistant strains of *Streptococcus pneumoniae* compared to the parent compound [16].

In this paper, we evaluate the antimicrobial activity of quaternary derivatives of cinchonidine and cinchonine by using disc diffusion and broth microdilution assay against a representative panel of Gram-positive and Gram-negative bacteria. For the most potent compounds, cytotoxicity was assessed on four different human cell lines as well as their influence on creating reactive oxygen species (ROS).

## 2. Materials and Methods

### 2.1. Synthesis of Quaternary Derivatives

The detailed synthetic protocols and spectral data of products have been previously reported [17]. Chemical structures of compounds are presented in Figure 1.

### 2.2. Antimicrobial Activity

Test microorganisms were obtained from the American Type Culture Collection (ATCC, Rockville, MD, USA) and Faculty of Science, University of Split, Croatia (FSST). Three Gram-negative bacteria were used: *Escherichia coli* (FSST 982), *Klebsiella pneumoniae* (FSST 011) and *Pseudomonas aeruginosa* (FSST 982); as well as four Gram-positive bacteria: *Bacillus cereus* (ATTC 11778), *Enterococcus faecalis* (ATCC 29212), *Staphylococcus aureus* (ATCC 25923), and *Clostridium perfringens* (FSST 4999). To reach optical density equivalent of 10^6^ colony-forming units (cfu/mL), bacterial strains were cultured overnight at 37 °C in tryptic soy broth (TSB). Compounds were dissolved in DMSO to obtain a stock solution of 10 mg/mL.

For disc diffusion assay, sterile Mueller–Hinton agar was dispensed in sterile petri dishes (90 mm diameter) and left at room temperature to solidify for 2 h. The paper discs (6 mm diameter) were placed on the agar surface and 50 µL of each compound was placed on an empty disc. Gentamicin was used as positive control and DMSO as solvent control. Petri dishes were left to stand for 20 min at room temperature before incubation at 37 °C for 24 h. The diameter of the inhibition zone was measured in mm and the experiment was repeated trice.

Broth microdilution assay was used to assess minimum inhibitory concentration (MIC) by standard two-fold serial microdilution assay according to Clinical and Laboratory Standards Institute. Gentamicin and cefotaxime were used as positive controls. A detailed description of experiments was previously reported [18,19].

### 2.3. MTT

To assess the influence of the compounds with antibacterial activity on human cell lines, EZ4U MTT assay (Biomedica, Vienna, Austria) was performed according to the manufacturer’s instructions. Briefly, after thawing, cell lines FB-35 (primary culture of foreskin fibroblasts), NDFH (normal human dermal fibroblasts) HaCaT (spontaneously transformed aneuploid immortal keratinocyte cell line from adult human skin), and HMEC-1 (human microvascular endothelial cell line) cell lines were cultivated in Dulbecco’s Modified Eagles Media (DMEM; Sigma-Aldrich, St. Louis, MO, USA) supplemented with 10% fetal calf serum (FCS). After growing to 80% of confluence, cells were trypsinized and plated at a density of 10,000 cells/well for 24 h. The next day, cells were treated with the compounds for additional 24 h. Compound with high antimicrobial activity, **CD-(*p*Br)**, was diluted to final concentrations of 1 µM, 5 µM, 10 µM, 50 µM, 100 µM, and 200 µM, while **CD-(*p*NO_2_), CD-(*p*Cl), CN-Met, CN-Bzl, CN-(*p*Cl),** and **CN-(*m*Br)** were diluted to 1 µM, 10 µM, and 100 µM, respectively. Control cells were not treated with anything, while vehicle control was DMSO in the concentration of the corresponding compound dilution. At the end of treatment, the colorless dye was added to each well and the color development was monitored by measuring absorbance at 450 nm, with 620 nm as a reference wavelength. All experiments were performed in technical and biological triplicates. The obtained data were analyzed by one-way ANOVA with Dunnett’s multiple comparisons test comparing each compound with the control.

### 2.4. Measurement of ROS, GSH and Catalase Activity

To determine the effect of the selected compounds on redox level in the cells, we measured levels of ROS as oxidative part, and GSH levels and catalase activity as antioxidative parts of the cell redox system. For these assays, the two compounds with the highest antibacterial activities were selected: **CD-(*p*Br)** and **CD-(*p*NO_2_)**.

To assess the influence of the compounds on ROS production, FB-35, HaCaT, and HMEC-1 cells were plated in black 96-microwell plates at a density of 10,000 cells/well in colorless DMEM with 10% FCS and left overnight to adhere. The next day, 2′,7′-dichlorofluorescin diacetate (DCF-DA) at a final concentration of 20 µM was added for 30 minutes in each well. After the end of incubation, media with DCF-DA was removed and fresh media alone, or with 1 µM, 10 µM, 100 µM of **CD-(*p*Br)**, and **CD-(*p*NO_2_)** was added to the cells. The ROS production was measured on fluorimeter/spectrometer plate reader Infinite 200 PRO (Tecan Group Ltd., Männedorf, Switzerland) at an excitation wavelength of 500 nm and emission detection at 530 nm.

GSH levels and catalase activity were assessed on FB-35, HaCaT, and HMEC-1 cell lines. Cells were plated at a density of 0.5 × 10^6^ cells/well overnight. The next day, cells were treated with 100 µM and 200 µM of **CD-(*p*Br)**, **CD-(*p*NO_2_)**, and equivalent concentrations of DMSO and were left overnight. After 24 h, cells were trypsinized and the dry pellet was stored at −80 °C until the GSH and catalase activity analysis. GSH analysis was performed after diluting samples to 0.03 mg/ml, and the addition of reaction mix (8 mM 5,5-dithio-bis-2-nitrobenzoic acid, 0.4 Units of GSH reductase, and 0.6 mM of NADPH in phosphate buffer 100 mM NaH_2_PO_4_, 5 mM EDTA, pH 7.4). The formation of yellow product, 2-nitro-5-thiobenzoic acid, was measured on a plate reader at 405 nm (Easy-Reader 400 FW; SLT Lab Instruments, GmbH, Salzburg, Austria). The catalase activity assay is based on the degradation of H_2_O_2_ by the catalase in the cell lysate. Catalase is the enzyme with one of the highest turnover numbers, making it the first to degrade H_2_O_2_. The reaction started with mixing 40 μL of cell lysate with 100 μL of 65 mM H_2_O_2_ for 5 minutes. The addition of 100 μL of 32.4 mM ammonium molybdate stopped the reaction. The intensity of the resulting yellow complex between ammonium molybdate and hydrogen peroxide was measured with a plate reader Multiskan EX (Thermo Electron Corporation, Shanghai, China) at 405 nm. Concentrations of hydrogen peroxide in a range from 0 to 75 mM were used as standards. One unit of catalase activity is defined as the amount of enzyme needed for degradation of 1 μmol of H_2_O_2_/min at 25 °C. Catalase activity was expressed as units per milligram of proteins in cell lysate (U mg^−1^).

### 2.5. Statistics

All experiments were performed in technical and biological triplicates. The obtained data were analyzed by one-way ANOVA with Dunnett’s multiple comparisons test comparing each compound with the control.

### 2.6. Principal Component Analysis

Multivariate analyses were conducted by a second-order tensor analysis tool known as principal component analysis (PCA) [20,21]. In PCA, the data matrix ***X*** of rank *r* is decomposed in the sum of *r* matrices $t_{i}p_{i}^{\tau }$ with rank 1 (Equation (1)):

(1) $$X=\overset{r}{\underset{i=1}{\sum }}t_{i}p_{i}^{\tau }$$

$t_{i}$ is a vector of scores and $p_{i}^{\tau }$ is a vector of loadings. PCA provides the best linear projection of multidimensional data by minimizing the least squares objective function. Scores are used for classification, while loadings can be used for the variability identification among the data. PCA development goes back to Beltrami [22] and Pearson [23], and the name was introduced by Harold Hotelling [24]. 

Disc diffusion assay and MIC data were arranged in the data matrix ***X***, and PCA on the covariance matrix was performed by our parallelized code for multi- and univariate analysis [25,26,27]. Extraction of eigenvectors was based on the NIPALS algorithm [28] and the obtained principal components were subsequently used as regressed variables.

### 2.7. Sampling of the Potential Energy Surfaces

Ab initio molecular dynamics simulations with *on-the-fly* calculations of forces were used as a sampling procedure for potential energy surfaces (PES). Equations were integrated using the velocity Verlet algorithm [29]. The PM7 method [30] implemented in MOPAC2016 [31] was used for calculation of forces in each point of the simulation. Molecular dynamics were conducted by using our *in-house* developed program *qcc* [32,33]. Phase space coverage was ensured by setting the initial temperature for Maxwell distribution of velocities to 773.15 K. During the simulation, temperature was controlled using the velocity scaling algorithm. Step size was 0.5 fs and a total of 5 million steps were computed for each compound. PES of compounds spanned in multidimensional space of Cartesius coordinates were evaluated by PCA, providing principal components for further regression.

### 2.8. Machine Learning Multivariate Linear Regression

Reduced spaces of multi-target antimicrobial activities were used as dependent variables for estimation of *Cinchona* alkaloids derivatives [17] activities. A panel of various Gram-positive and Gram-negative bacteria provided activity data whose principal components were extracted by the second-order tensor decomposition. These principal components were regressed on the theoretically computed energy fingerprints of all compounds by performing extensive machine learning (ML).

The ML procedure was applied for the generation of all possible multivariate linear regression models with a linear combination of original variables as well as their higher-order polynomial terms. Multivariate linear regression was performed using the following expression for matrices of coefficients B calculated by singular value decomposition:

(2) $$B={\left(X^{\tau }X\right)}^{-1}X^{\tau }Y$$

where X and Y are the matrices of independent and dependent variables, respectively. Every possible regression model of antimicrobial activity dependent on molecular dynamics data was built and thoroughly validated by the *leave-one-out cross-validation* technique (LOO-CV). The models were inspected up to the sixth order for 2D models and up to the fourth order for 3D models, and the total numbers of investigated models were 134,217,728 and 17,179,869,184, respectively. The most optimal representations were selected based on the adjusted and predicted *R^2^* values, LOO-CV mean squared error, as well as the number of variables in the models.

## 3. Results and Discussion

### 3.1. Synthesis

A series of differently substituted quaternary ammonium salts of **CD**s and their corresponding quasi-enantiomeric **CN**s were synthesized by reaction of commercially available cinchonidine or cinchonine and alkyl or arylalkyl halides in refluxing toluene by published procedures [17]. Compounds **CD-Met** and **CN-Met** were prepared in reaction of the appropriate alkaloid with methyl iodide, **CD-Bzl** and **CN-Bzl** with benzyl bromide, and other compounds with appropriate *meta*- and *para*-substituted benzyl bromides, different in size and electronic properties. Compounds were characterized by standard analytical methods (IR, 1D and 2D NMR, MS, CHN analysis).

### 3.2. Antimicrobial Activity

Unmodified parent alkaloids **CD** and **CN** as well as prepared quaternary derivatives of quasi-enantiomers were screened for antimicrobial activity on different Gram-positive and Gram-negative bacteria by disc diffusion method. Activities of the target compounds were expressed as the mean diameter of the measured inhibition zone (mm) against selected microorganisms along with the activity of the reference compound gentamicin, Table 1.

Most of the quaternary *Cinchona* alkaloid derivatives showed potent and broad-spectrum activity against selected clinically important pathogens with mean diameters of inhibition zone of compounds in the range from 6.4 ± 0.9 to 28.5 ± 2.8 mm. Tested compounds demonstrated antibacterial effects against both Gram-positive and Gram-negative bacteria. Generally, quaternary derivatives of cinchonidine showed more antibacterial activity than corresponding quaternary derivatives of quasi-enantiomer cinchonine. Interestingly, most of the tested compounds were very effective on *E. coli* and *P. aeruginosa*, showing potential use for P. aeruginosa which is on the critical list for resistance [1]. In detail, considerable zones of growth inhibition were observed for two tested strains of Gram-negative bacteria, *E. coli* (from 10.5 ± 1.1 to 25.7 ± 2.7 mm) and *P. aeruginosa* (from 7.4 ± 1.9 to 28.5 ± 2.8 mm). Compounds **CD-(*p*Br)** and **CD-(*p*NO_2_)**, which have bromine atom or nitro group in *para* position on the benzene ring, showed the most potent activity toward *E. coli* with the mean inhibition diameters of 25.7 ± 2.7 mm for **CD-(*p*Br)** and 23.1 ± 1.4 mm for **CD-(*p*NO_2_)**.

Quaternary derivatives of **CD** and **CN** were then tested against the same panel of Gram-positive and Gram-negative bacteria to determine MIC values by a broth microdilution method. The results of antimicrobial assays are summarized in Table 2.

All prepared quaternary derivatives demonstrated potent and broad-spectrum activities against selected microorganisms with MIC values in the range of 1.56 to 125.00 μg/mL. Compounds **CD-(*p*Br)** and **CD-(*p*NO_2_)** were found to possesses not only strong and very strong activity against all tested Gram-positive bacteria (MIC values in the range of 6.25–12.00 μg/mL) but also very strong activity against all tested Gram-negative bacteria (MIC values 6.25 μg/mL), which are fivefold more potent than gentamicin toward *E. coli* and tenfold more potent than gentamicin toward *P. aeruginosa,* thereby supporting the disc diffusion assay. Compound **CD-(*p*Cl)** showed very strong activity against *E. coli* (MIC value 6.25 μg/mL) which is fivefold more active than gentamicin. Other quaternary derivatives of **CD** were also quite active toward *E. coli,* with MIC values in the range of 25.00 to 50.00 μg/mL, while quaternary derivatives of quasi-enantiomers in **CN** series did not show similar activity against *E. coli*. Compounds **CD-(*p*Br)** and **CN-Bzl** showed very strong activity against *P. aeruginosa* with a MIC value of 1.56 μg/mL which is fortyfold more active than gentamicin and tenfold more active than cefotaxime.

All prepared quaternary derivatives of **CD** exhibited strong activity toward *P. aeruginosa* (MIC values in the range of 1.56 to 50.00 μg/mL) except unmodified parent alkaloid **CD** (MIC value 125.00 μg/mL). Most of the prepared quaternary derivatives of **CN** displayed moderate activity toward *P. aeruginosa* with MIC values up to 125.00 μg/mL, but some of them have very strong activity with MIC values from 3.12 μg/mL to 12.50 μg /mL, which is twentyfold more active than gentamicin and fivefold more active than cefotaxime. Based on the acquired results, the stereochemistry of the antibacterial compound is important to some extent for the bioactivity toward *P. aeruginosa*. Compounds **CD-(*p*Br)** and **CN-Bzl** are 32 times more active than their *quasi*-enantiomers **CN-(*p*Br)** and **CD-Bzl**.

Taking these two assays together, **CD-(*p*Br)** and **CD-(*p*NO_2_)** were effective for both Gram-positive and Gram-negative bacteria, indicating that the tested compounds do not inhibit cell wall synthesis. A possible target of these cinchonine derivatives might be the bacterial ATP synthase, which also appears to be the target for another cinchone derivate [16]. Certainly, the mechanism should be further investigated, especially due to reactivity toward *P. aeruginosa*.

### 3.3. Cytotoxicity

**Figure 2:**
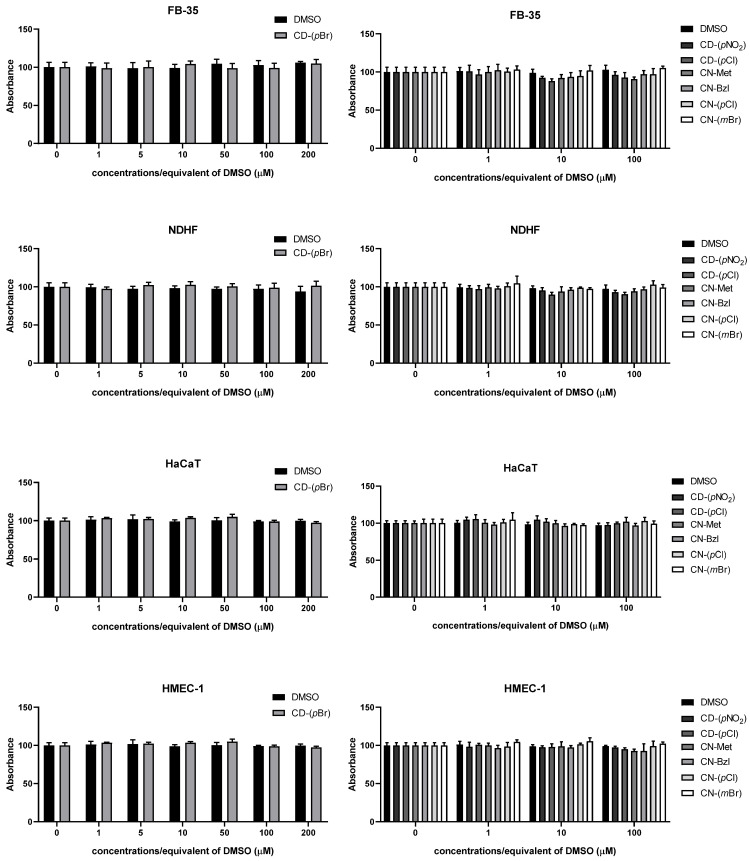
Effects of compounds on FB-35 (normal foreskin fibroblasts), NDFH (normal human dermal fibroblasts), HaCat (keratinocytes), and HMEC-1 (microvascular endothelial cell line) viability. *p* > 0.05.

Compounds with strong activity toward tested bacterial strains were further evaluated for cytotoxicity on four different human cell lines (Figure 2). There was no change in cell viability in any of the tested compounds compared to both control and vehicle control. For compound **CD-(*p*Br)**, testing a wider concentration range showed no changes in cell viability (Figure 2).

### 3.4. Effects of the Compounds on Cellular Reactive Oxygen Species and Antioxidative Defense

**Figure 3:**
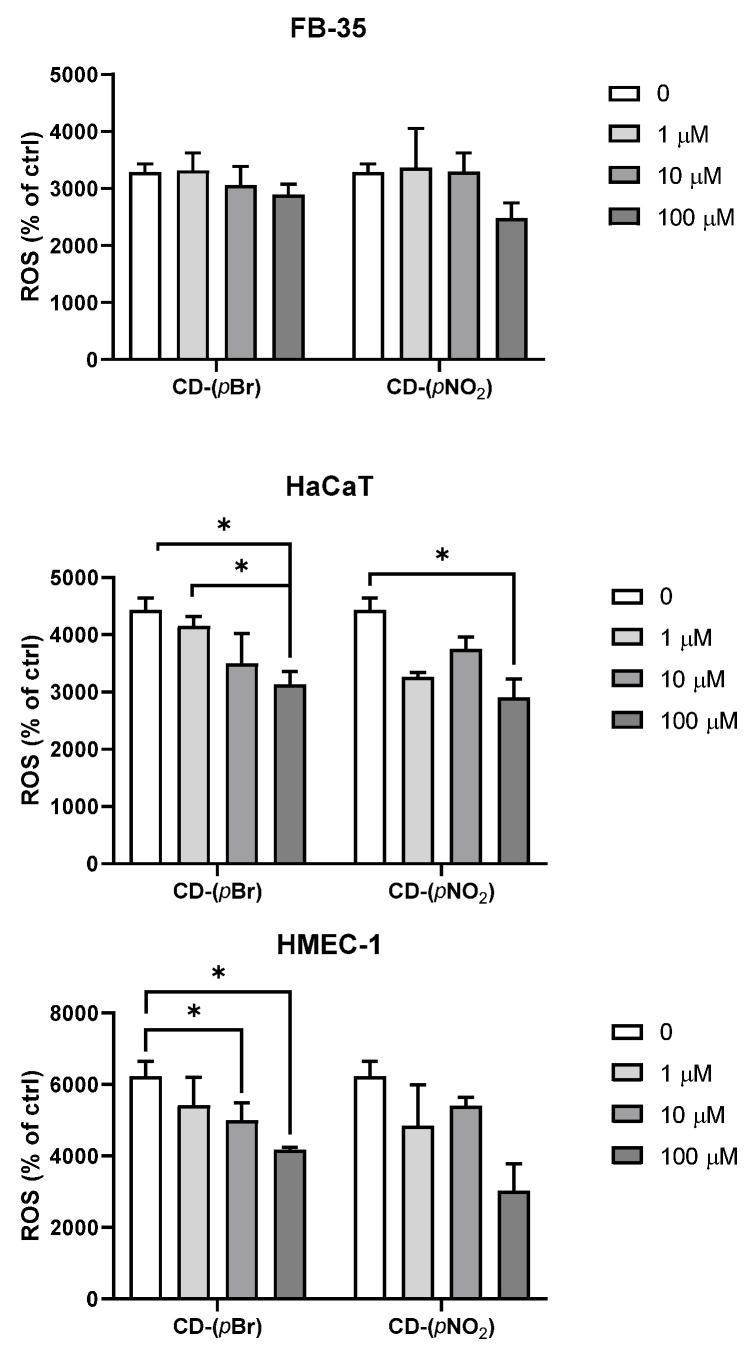
Effects of compounds **CD-(pBr)** and **CD-(*p*NO_2_)** on ROS levels in FB-35, HaCaT, and HMEC-1 cell lines after 2 h treatment.* *p* > 0.05.

After MTT showed no difference in cell viability, we assessed the influence of the compounds with strong antimicrobial activity on ROS production. Although there are differences in ROS levels after treatments (Figure 3), these are not statistically significant.

Effects of the **CD-(*p*Br)** and **CD-(*p*NO_2_)** compounds on cellular ROS levels are cell line-specific. While the two compounds did not significantly affect ROS levels in FB-35 fibroblast, **CD-(*p*Br)** decreased levels of ROS in HaCaT (control vs. 100 µM *p* = 0.044; and 1 µM vs. 100 µM *p* = 0.011) and in HMEC-1 cells (control vs. 10 µM or 100 µM, *p* = 0.017 and *p* = 0.029, respectively). The **CD-(*p*NO_2_)** compound decreased ROS levels only in the HaCaT cell line (control vs. 100 µM, *p* = 0.013).

Despite obvious differences between cell lines, both tested compounds did not show statistical differences at the tested concentrations.

**Figure 4:**
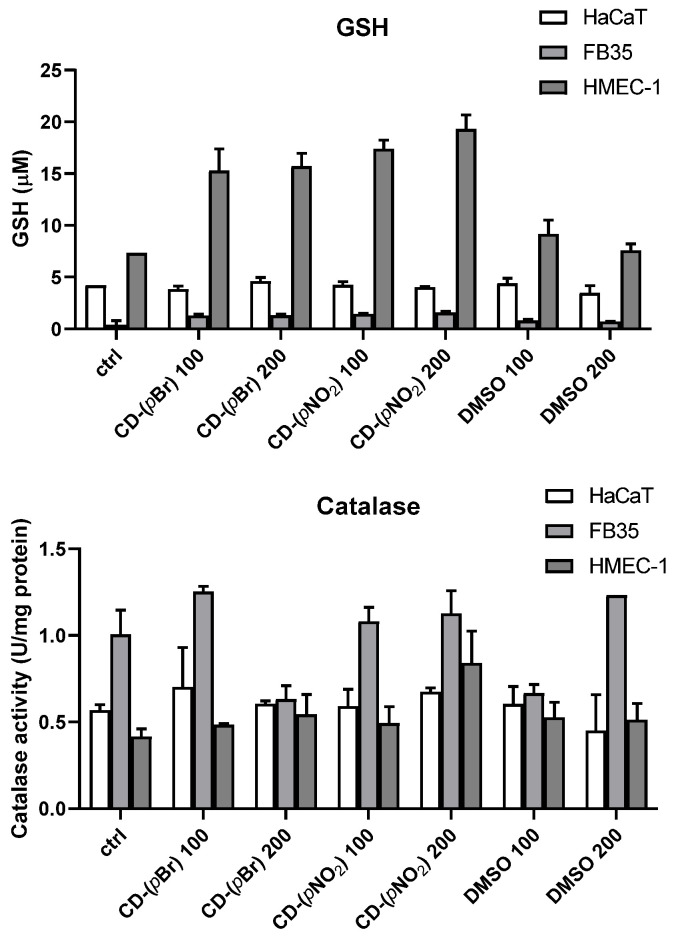
Effects of **CD-(*pBr*)** and **CD-(*p*NO_2_)** on GSH levels in FB-35, HaCaT, and HMEC-1.

The influence of the compound **CD-(*p*Br)**, which had the most potent antibacterial activity, on GSH levels was assessed on FB-35, HaCaT, and HMEC-1 cell lines. GSH levels in all tested cell lines were not affected (Figure 4). Likewise, catalase activity was not affected by the compound **CD-(*p*Br)**.

### 3.5. PCA Analysis and Activity/PES Model

**Figure 5:**
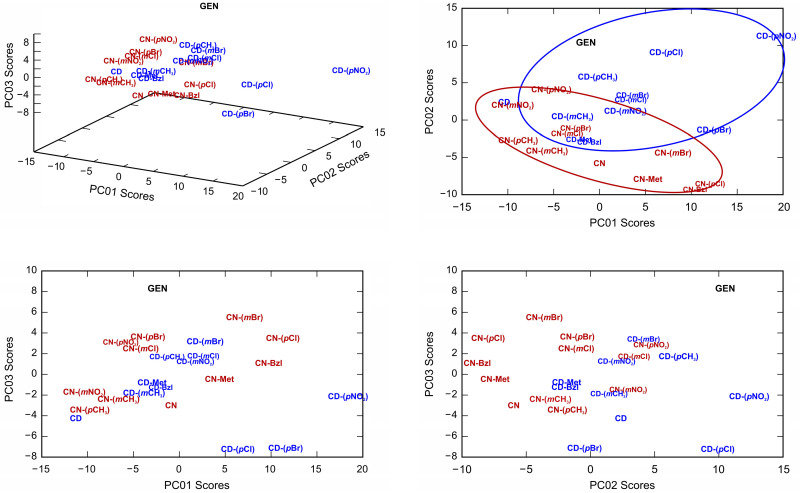
Classification of compounds based on values calculated by PCA performed on the mean-centered covariance matrix of their disc diffusion assay values.

**Figure 6:**
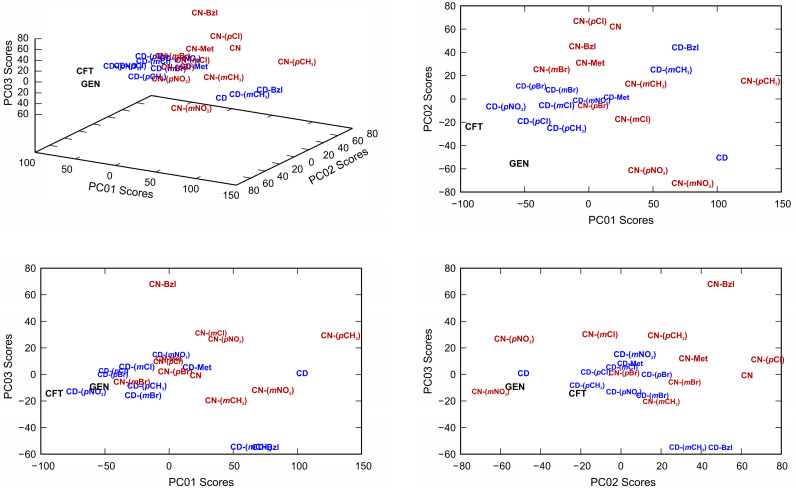
Classification of compounds based on values calculated by PCA performed on the mean-centered covariance matrix of their MIC values.

To classify investigated compounds according to their activities set side-by-side with the tested standard antibiotics (Table 1 and Table 2), we performed PCA on the disc diffusion assay data and MIC values [23]. In both cases, the first three principal components explained more than 82% of the total variance among the data, ensuring the proper description of the activities in this reduced three-dimensional space. Effectively, the seven-dimensional space of multi-target antimicrobial activities was reduced to only three dimensions and retained the majority of the information present in the original data. An additional advantage of using data space reduced to three dimensions is the possibility of visualization and presenting graphical layouts. Therefore, we used 3D models, presented in Figure 5 and Figure 6 (together with all 2D projections), to perform classification and identification of principal component directions that are the most important for evaluating activities. From the classification model for disc diffusion assay data presented in Figure 5, it is evident that the second principal component describes the antimicrobial activity. Using the position of compounds in this new reduced space, several promising candidates were found, e.g., **CD-(*p*NO_2_)** was having a score of 11.28 which is even higher than for **GEN** (10.36, Figure 5). In the group of promising candidates, there was also **CD-(*p*Cl)** with the score of 9.09.

According to the MIC values, the first principal component in the negative direction was the most important in describing the antimicrobial activity of the compounds (Figure 6). Interpretation of principal components is invariant to the sign of the component (the component with the negative sing is still an eigenvector of covariance matrix), so the fact that some compounds are shifted along this axis in the negative direction (or in the positive) is not important for further analysis. Likely candidates were identified as **CD-(*p*NO_2_)**, **CD-(*p*Br)**, and **CD-(*p*Cl)**.

To establish a connection between antimicrobial data and calculated potential energy surfaces of the compounds, an activity/PES model was created by using the first two and first three principal components of the reduced PES data and the selected principal components from the disc diffusion assay and MIC data. As identified in the classification models obtained by PCA, for disc diffusion data the factor scores along the second principal component were regressed on the first two and the first three principal components of the reduced PES data. For MIC data, the factor scores along the first principal component were regressed on the first two and the first three principal components of the reduced PES data.

An extensive machine learning procedure for multivariate linear regression was performed. The objective of the machine learning was the determination of the best possible regression model that can explain compounds’ multi-target antimicrobial activities regressed on the theoretically computed potential energy surfaces. All possible regression models were generated, and the B-matrices of coefficients (Equation 2) were determined. Each model was validated using the *leave-one-out cross-validation*. The best regression models were selected based on the adjusted *R^2^* and predicted *R^2^* values, LOO-CV mean squared error, as well as the number of variables in the models [34].

**Figure 7:**
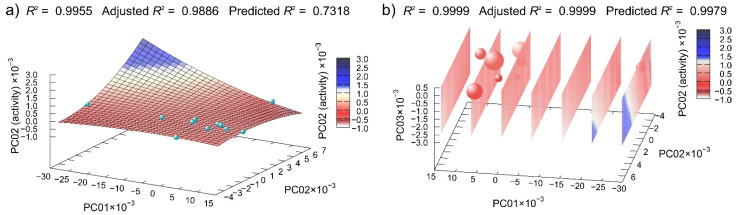
Machine learning determined best multivariate regression models of **CD** derivatives disc diffusion assay data dependent on the (**a**) first two and (**b**) first three principal component of compounds potential energy surfaces. (In (**b**), spheres represent points in 3D-reduced space, and the planes are cuts of polynomial regression model; for easier interpretation, the fourth dimension is represented redundantly with the color and size of the spheres.)

**Figure 8:**
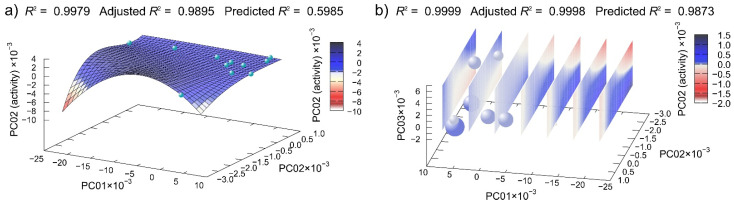
Machine learning determined best multivariate regression model of **CN** derivatives disc diffusion assay data dependent on the (**a**) first two and (**b**) first three principal component of compounds potential energy surfaces. (In (**b**), spheres represent points in 3D-reduced space, and the planes are cuts of polynomial regression model; for easier interpretation, the fourth dimension is represented redundantly with the color and size of the spheres.)

Disc diffusion assay data for **CD** derivatives were regressed and the best calculated 2D and 3D regression models are presented in Figure 8. Despite the very high valued of *R^2^* and adjusted *R^2^* in 2D model (Figure 7a), the value of predicted *R^2^* had a lower value of only 0.7318, indicating the overfitting. Due to this reason, we also calculated the 3D regression model. The 3D model had an excellent value of predicted *R^2^* = 0.9979 (Figure 7b), confirming the validity of this model. 

For derivatives of **CN**, the best 2D and 3D regression models are presented in Figure 8. Although the *R*^2^ in the 2D model had value of 0.9979 (Figure 8a), the value of predicted *R*^2^ was 0.5985, again indicating the overfitting. As in the previous case, we extended the model to three dimensions, producing the 3D model, which had very good value of predicted *R^2^* = 0.9873 (Figure 8b). 

**Figure 9:**
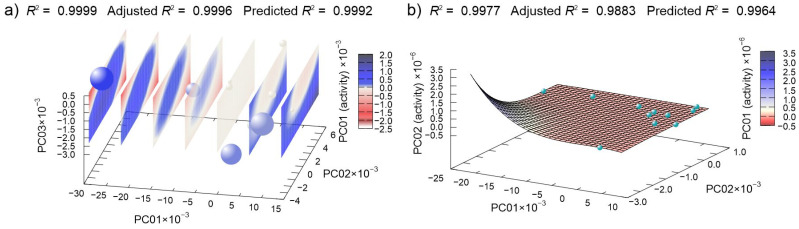
Machine learning determined best multivariate regression models of (**a**) **CD** derivatives and (**b**) **CN** derivatives MIC data dependent on the principal component of compounds potential energy surfaces. (In (**a**), spheres represent points in 3D-reduced space, and the planes are cuts of polynomial regression model; for easier interpretation, the fourth dimension is represented redundantly with the color and size of the spheres.)

Regression of MIC data provided models for derivatives of **CD** (Figure 9a) and derivatives of **CN** (Figure 9b). In both cases, the best established models have very high values of predicted *R*^2^, confirming the quality of the models.

An established activity/PES model can be used for the prediction of antimicrobial activities for new compounds based solely on the reduced space of compounds potential energy surfaces. The models will work good for similar compounds, i.e., **CD** or **CN** derivatives, while for the different type of compounds with significantly different chemical structure, one cannot expect the models to work.

## 4. Conclusions

The biological activity of unmodified alkaloids **CD** and **CN** and their *N*-alkyl and *N*-aryl quaternary derivatives was determined. Compounds **CD-(*p*Br)**, **CD-(*p*NO_2_)**, **CD-(*p*Cl)**, **CN-CH_3_**, **CN-Bzl**, **CN-(*p*NO_2_),** and **CN-(*p*Cl)** showed strong antimicrobial activity toward the tested representative panel of bacteria, especially the emerging pathogen *Pseudomonas aeruginosa* (the lowest MIC value was 1.56 μg/mL). Compounds do not show a toxic effect or an effect on the production of reactive oxygen species in different human cell lines. An extensive machine learning procedure for the generation of multivariate linear regression models with a linear combination of original variables as well as their higher-order polynomial terms was performed. Among all statistically possible regression models, the best possible models with predicted *R^2^* > 0.98 were determined. This activity/PES model can be used for accurate prediction of activities for new **CD** and **CN** derivatives based solely on their potential energy surfaces, which will enable wider screening and faster search for new potential leads. Based on obtained results, *N*-quaternary derivatives of *Cinchona* alkaloids proved to be an excellent scaffold for further optimization of novel antibiotic species.

## Figures and Tables

**Table 1:** Antimicrobial activity of **CD** and **CN** derivatives against a panel of Gram-positive and Gram-negative bacterial strains determined by disc diffusion assay. According to the sizes of the inhibitory zone (including the diameter of a disc), the antimicrobial activity is graded as inactive (0–9 mm); mildly active (10–15 mm); moderately active (16–20 mm); and highly active (≥21 mm). All values are expressed as mean ± SD of three parallel measurements (*n* = 3).

Compounds	Diameters of the Inhibition Zone (mm) ^a^						
	Gram-Positive Bacteria				Gram-Negative Bacteria		
	*B. cereus*	*E. faecalis*	*S. aureus*	*C. perfringens*	*E. coli*	*K. pneumoniae*	*P. aeruginosa*
**CD**	10.9 ± 1.7	14.2 ± 0.9	13.3 ± 0.6	14.6 ± 0.7	**17.2 ± 1.4**	10.2 ± 1.1	6.4 ± 0.9
**CD-Met**	12.7 ± 1.1	11.2 ± 1.4	15.7 ± 1.7	14.2 ± 1.3	**14.8 ± 1.2**	12.4 ± 0.9	**15.8 ± 2.1**
**CD-Bzl**	9.4 ± 0.5	**15.7 ± 1.9**	18.5 ± 1.2	8.4 ± 2.2	**15.6 ± 1.6**	16.7 ± 1.6	**16.3 ± 1.7**
**CD-(*p*Br)**	16.6 ± 1.4	**15.6 ± 2.3**	17.4 ± 1.8	15.6 ± 0.9	**25.7 ± 2.7**	14.7 ± 0.6	**25.7 ± 2.4**
**CD-(*p*CH_3_)**	15.8 ± 2.2	**16.8 ± 0.9**	17.8 ± 2.3	18.1 ± 0.9	**14.8 ± 0.9**	15.8 ± 0.9	**11.8 ± 0.9**
**CD-(*p*NO_2_)**	**22.8 ± 0.8**	**27.4 ± 1.7**	17.4 ± 0.3	21.8 ± 0.9	**23.1 ± 1.4**	**26.1 ± 2.1**	**25.1 ± 1.6**
**CD-(*p*Cl)**	15.3 ± 1.0	**16.3 ± 1.3**	15.5 ± 1.7	**23.3 ± 1.7**	**24.5 ± 1.8**	19.5 ± 1.3	**17.2 ± 1.2**
**CD-(*m*Br)**	14.8 ± 1.3	**19.6 ± 1.2**	21.4 ± 0.7	13.8 ± 0.6	**14.6 ± 1.2**	17.6 ± 1.3	**16.3 ± 1.9**
**CD-(*m*CH_3_)**	9.8 ± 1.0	**17.3 ± 2.3**	20.3 ± 1.8	10.3 ± 1.1	**17.7 ± 0.7**	12.7 ± 0.6	**12.1 ± 2.7**
**CD-(*m*Cl)**	15.0 ± 1.6	**16.0 ± 1.8**	19.0 ± 1.4	16.0 ± 0.8	**15.2 ± 2.4**	18.2 ± 0.7	**17.2 ± 1.3**
**CD-(*m*NO_2_)**	15.3 ± 0.6	**16.2 ± 2.5**	19.2 ± 1.5	17.2 ± 1.3	**15.4 ± 1.8**	12.4 ± 3.1	**17.4 ± 1.4**
**CN**	13.2 ± 0.9	14.2 ± 1.9	13.2 ± 1.8	10.2 ± 1.5	**16.3 ± 1.2**	12.6 ± 1.4	**19.2 ± 2.7**
**CN-Met**	12.4 ± 1.0	13.4 ± 1.5	16.4 ± 1.2	11.4 ± 1.1	**14.4 ± 2.1**	13.4 ± 1.0	**25.4 ± 3.2**
**CN-Bzl**	**22.7 ± 1.9**	9.6 ± 1.6	13.4 ± 1.6	12.1 ± 1.5	**15.6 ± 2.5**	13.9 ± 0.6	**27.6 ± 3.2**
**CN-(*p*Br)**	15.7 ± 1.8	**17.7 ± 0.9**	16.2 ± 1.7	10.7 ± 1.3	11.2 ± 0.7	12.1 ± 2.3	**13.2 ± 0.5**
**CN-(*p*CH_3_)**	8.8 ± 1.3	9.2 ± 1.7	10.8 ± 1.4	11.8 ± 2.6	**13.4 ± 1.5**	14.2 ± 1.5	**10.1 ± 1.3**
**CN-(*p*NO_2_)**	13.3 ± 1.6	12.9 ± 1.7	15.7 ± 2.5	19.4 ± 2.1	10.5 ± 1.2	14.7 ± 1.2	**10.2 ± 2.1**
**CN-(*p*Cl)**	**21.5 ± 1.5**	14.5 ± 1.7	17.5 ± 1.3	10.5 ± 1.1	**14.5 ± 1.3**	13.5 ± 1.6	**28.5 ± 2.8**
**CN-(*m*Br)**	16.6 ± 1.3	**17.2 ± 1.3**	20.6 ± 2.2	12.6 ± 1.4	11.6 ± 2.3	14.6 ± 1.1	**24.6 ± 3.0**
**CN-(*m*CH_3_)**	7.7 ± 1.6	**16.7 ± 0.6**	11.5 ± 1.2	12.1 ± 1.3	11.9 ± 1.5	10.2 ± 0.9	**14.4 ± 1.7**
**CN-(*m*Cl)**	15.2 ± 0.6	13.2 ± 2.3	16.2 ± 1.6	15.2 ± 3.2	**12.2 ± 0.9**	9.2 ± 1.4	**13.9 ± 2.1**
**CN-(*m*NO_2_)**	9.4 ± 1.5	12.1 ± 1.3	15.4 ± 2.4	15.7 ± 2.2	**14.4 ± 2.3**	11.2 ± 2.0	7.4 ± 1.9
**GEN** ^b^	**18.2 ± 0.7**	**14.6 ± 1.4**	**23.9 ± 0.9**	**21.7 ± 0.4**	**11.5 ± 0.9**	**18.8 ± 0.6**	**9.7 ± 1.4**

^a^ Diameter of inhibition zone (values in mm) around the disc: 200 μg/disc. ^b^ Gentamicin standard antibiotic disc (15 μg/disc).

**Table 2:** Determined MIC values for **CD** and **CN** quaternary derivatives, gentamicin (GEN) and cefotaxime (CFT) against a panel of Gram-positive and Gram-negative bacterial strains. No bioactivity was defined as a MIC > 1000 μg/mL, mild bioactivity as a MIC in the range 512–1000 μg/ mL, moderate bioactivity as a MIC in the range 128–512 μg/mL, good bioactivity as a MIC in the range 32–128 μg/mL, strong bioactivity as a MIC in the range 10–32 μg/mL, and very strong bioactivity as a MIC < 10 μg/mL.

Compounds	MIC (µg/mL)						
	Gram-Positive Bacteria				Gram-Negative Bacteria		
	*B. cereus*	*E. faecalis*	*S. aureus*	*C. perfringens*	*E. coli*	*K. pneumoniae*	*P. aeruginosa*
**CD**	100.00	50.00	50.00	50.00	25.00	100.00	125.00
**CD-Met**	50.00	50.00	25.00	50.00	25.00	50.00	50.00
**CD-Bzl**	100.00	25.00	25.00	125.00	50.00	50.00	50.00
**CD-(*p*Br)**	25.00	25.00	25.00	25.00	**6.25**	25.00	**1.56**
**CD-(*p*CH_3_)**	25.00	12.50	25.00	25.00	25.00	25.00	50.00
**CD-(*p*NO_2_)**	12.50	**6.25**	12.50	12.50	**6.25**	**6.25**	**6.25**
**CD-(*p*Cl)**	25.00	25.00	25.00	**6.25**	**6.25**	12.50	25.00
**CD-(*m*Br)**	25.00	12.50	12.50	50.00	50.00	25.00	25.00
**CD-(*m*CH_3_)**	100.00	25.00	12.50	100.00	25.00	50.00	50.00
**CD-(*m*Cl)**	25.00	25.00	25.00	25.00	50.00	25.00	25.00
**CD-(*m*NO_2_)**	50.00	50.00	25.00	25.00	25.00	50.00	25.00
**CN**	50.00	50.00	50.00	100.00	25.00	50.00	12.50
**CN-Met**	50.00	50.00	25.00	50.00	50.00	50.00	**3.12**
**CN-Bzl**	12.50	100.00	50.00	50.00	25.00	50.00	**1.56**
**CN-(*p*Br)**	25.00	25.00	25.00	50.00	50.00	50.00	50.00
**CN-(*p*CH_3_)**	100.00	100.00	100.00	100.00	50.00	50.00	100.00
**CN-(*p*NO_2_)**	50.00	50.00	25.00	12.50	100.00	50.00	100.00
**CN-(*p*Cl)**	12.50	50.00	25.00	100.00	50.00	50.00	**3.12**
**CN-(*m*Br)**	25.00	25.00	12.50	50.00	50.00	25.00	**3.12**
**CN-(*m*CH_3_)**	100.00	25.00	50.00	50.00	50.00	50.00	25.00
**CN-(*m*Cl)**	50.00	50.00	25.00	25.00	50.00	100.00	50.00
**CN-(*m*NO_2_)**	100.00	50.00	25.00	25.00	25.00	50.00	125.00
**GEN**	**4.00**	**4.00**	**1.00**	**0.50**	32.00	**8.00**	64.00
**CFT**	**0.25**	**0.50**	**0.50**	**0.10**	**0.50**	**0.50**	16.00

## Data Availability

The data for this manuscript is available from correspondence author.
